# Multi-species bacterial biofilm and intracellular infection in otitis media

**DOI:** 10.1186/1471-2431-11-94

**Published:** 2011-10-24

**Authors:** Ruth B Thornton, Paul J Rigby, Selma P Wiertsema, Pierre Filion, Jennifer Langlands, Harvey L Coates, Shyan Vijayasekaran, Anthony D Keil, Peter C Richmond

**Affiliations:** 1 School of Paediatrics and Child Health, The University of Western Australia, Perth, Western Australia, Australia; 2 Vaccine Trials Group, Telethon Institute for Child Health Research, Subiaco, Western Australia, Australia; 3 Centre for Microscopy, Characterisation and Analysis, The University of Western Australia, Perth, Western Australia, Australia; 4 Department of Pathology, PathWest Laboratory Medicine WA, Sir Charles Gairdner Hospital, Western Australia, Australia; 5 Department of Otolaryngology, Head and Neck Surgery, Princess Margaret Hospital for Children, Perth, Western Australia, Australia; 6 Department of Otolaryngology, Head and Neck Surgery, The University of Western Australia, Perth, Western Australia, Australia; 7 Department of Microbiology, PathWest Laboratory Medicine WA, Princess Margaret Hospital for Children, Perth, Western Australia, Australia

**Keywords:** Bacterial biofilm, intracellular infection, otitis media, fluorescent in situ hybridisation, transmission electron microscopy

## Abstract

**Background:**

Bacteria which are metabolically active yet unable to be cultured and eradicated by antibiotic treatment are present in the middle ear effusion of children with chronic otitis media with effusion (COME) and recurrent acute otitis media (rAOM). These observations are suggestive of biofilm presence or intracellular sequestration of bacteria and may play a role in OM pathogenesis. The aim of this project is to provide evidence for the presence of otopathogenic bacteria intracellularly or within biofilm in the middle ear mucosa of children with COME or rAOM.

**Methods:**

Middle ear mucosal biopsies from 20 children with COME or rAOM were examined for otopathogenic bacteria (either in biofilm or located intracellularly) using transmission electron microscopy (TEM) or species specific fluorescent *in situ *hybridisation (FISH) and confocal laser scanning microscopy (CLSM). One healthy control biopsy from a child undergoing cochlear implant surgery was also examined.

**Results:**

No bacteria were observed in the healthy control sample. In 2 of the 3 biopsies imaged using TEM, bacteria were observed in mucus containing vacuoles within epithelial cells. Bacterial species within these could not be identified and biofilm was not observed. Using FISH with CLSM, bacteria were seen in 15 of the 17 otitis media mucosal specimens. In this group, 11 (65%) of the 17 middle ear mucosal biopsies showed evidence of bacterial biofilm and 12 demonstrated intracellular bacteria. 52% of biopsies were positive for both biofilm and intracellular bacteria. At least one otopathogen was identified in 13 of the 15 samples where bacteria were present. No differences were observed between biopsies from children with COME and those with rAOM.

**Conclusion:**

Using FISH and CLSM, bacterial biofilm and intracellular infection with known otopathogens are demonstrated on/in the middle ear mucosa of children with COME and/or rAOM. While their role in disease pathogenesis remains to be determined, this previously undescribed infection pattern may help explain the ineffectiveness of current treatment strategies at preventing or resolving COME or rAOM.

## Background

The important role of bacteria in otitis media (OM) pathogenesis has long been acknowledged, however the aetiology of recurrence and persistence of this condition is not well understood. Many characteristics suggest that chronic otitis media with effusion (COME) and recurrent acute otitis media (rAOM) are biofilm related [1-3]. Biofilms are defined as clusters of bacteria embedded in a polymeric matrix with increased resistance to antibiotics and host defence mechanisms when compared to their "planktonic" or "free floating" counterparts [4]. While biofilm has been demonstrated in OM animal models, [5,6] there is limited data available on biofilm formation in the middle ears of children with OM [7,8].

Our group has previously demonstrated intracellular infection of the middle ear mucosa in a small number of children with COME using transmission electron microscopy (TEM) [9]. However the bacterial species within these epithelial cells were not identified. Although it is known that some otopathogenic bacteria, including nontypeable *Haemophilus influenzae*, *Moraxella catarrhalis *and *Streptococcus pneumoniae*, are able to invade and survive within cells *in vitro *[10-15] and in adenoidal cells [16,17] it is unclear if this occurs *in vivo *in the middle ears of children with OM. This has important implications with regards to treatment, as the β-lactam antibiotics often used to treat OM episodes show poor penetration of cells and thus poor efficacy against bacteria sequestered intracellularly [12,18].

Despite polymicrobial biofilms being common [19], to date most reports (with the exception of Hall-Stoodley *et al *[8]) have not attempted to identify bacterial species present in the middle ear or have limited their analysis to a single species [20]. It is important to determine the presence of these otopathogenic species to determine the contribution of intracellular bacteria and biofilm formation to disease pathogenesis and for the development of new treatment strategies to combat this common childhood disease.

We hypothesise that multispecies bacterial biofilm and intracellular infection are both present in the middle ear mucosa of children with rAOM and COME. We believe this contributes to the chronic and recrudescent infections observed in these children. To investigate this hypothesis we used TEM and confocal laser scanning microscopy (CLSM) combined with bacterial-specific fluorescent *in situ *hybridisation (FISH) on middle ear biopsies taken from children undergoing ventilation tube insertion for rAOM and/or COME.

## Methods

### Patient population

Twenty children aged between 0 and 10 years were recruited at time of admission for ventilation tube insertion for a history of COME and/or rAOM through public hospitals in Perth, Australia. Due to public hospital waiting lists, several children were recruited between AOM or OME episodes.

COME was defined as the presence of a middle ear effusion without the symptoms or signs of suppurative infection for longer than 3 months [21]. rAOM was defined as having at least 3 acute OM presentations within a 6 month period or 4 or more episodes in a 12 month period, between which clinical symptoms resolved. Exclusion criteria included known immunodeficiency, chromosomal or craniofacial disorders.

One healthy mucosal control subject was recruited at time of cochlear implantation surgery. This child had no history of chronic or recurrent middle ear disease, nor any exclusion criteria for cases.

Clinical data including immunisation status was collected using parental questionnaires and medical records. Written informed consent was obtained from parents or guardians prior to enrolment in the study. Approval for this study was obtained from the Princess Margaret Hospital for Children, Armadale-Kelmscott and Osborne Park Hospital Ethics Committees.

### Specimen acquisition and tissue preparation

General laryngeal mask anaesthesia was used during the surgical procedure and utilizing the operating microscope, an anterior-inferior myringotomy incision was made. If middle ear effusion (MEE) was present, this was collected utilizing a sterile tympanostomy trap. MEE specimens were transported on ice to the PathWest clinical microbiology laboratory at Princess Margaret Hospital for Children for detection of bacteria using culture and PCR. Utilizing micro cup forceps via the myringotomy incision, middle ear mucosa biopsies of approximately 1 mm^3 ^were obtained from the promontory near the eustachian tube. One biopsy was obtained for assessment by microscopy for each child. Biopsies for TEM imaging were fixed in 2.5% glutaraldehyde in phosphate buffer (pH 7.4) until processing. Biopsies for FISH and CLSM were fixed overnight in 4% paraformaldehyde in phosphate buffered saline (PBS) (pH 7.2), washed three times with PBS and stored in 50% PBS/Ethanol at -20°C prior to hybridisation.

### Bacterial cultures

MEE samples were assayed using standard diagnostic culture techniques. Primary inoculations of MEE samples were made on blood agar, cysteine lactose electrolyte deficient agar, Filde's agar and colistin nalidixic acid (10 mg/L) blood agar plates. Plates were incubated at 35°C in 5% CO_2 _and inspected for growth at 24 and 48 hours. In addition, inoculated anaerobic blood agar with vancomycin (2.5 mg/L) and nalidixic acid (10 mg/L) and colistin nalidixic acid blood agar were incubated anaerobically for 7 days with inspection at 48 hours and 7 days. All predominant bacteria were recorded including normal nasopharyngeal flora.

### Pneumolysin PCR

Pneumolysin (Ply) is a toxin present in *Streptococcus pneumoniae *and several closely related commensal bacteria including *Streptococcus mitis, Streptococcus oralis *and *Streptococcus pseudopneumoniae*. Genomic DNA was isolated from the MEE specimens using the Roche Total Nucleic Acid Extraction kits (Roche Diagnostics) following manufacturers instructions. Real time PCR to detect the *ply *gene was performed on the extracted DNA as previously described [22].

### Microscopic examination

#### Transmission electron microscopy

Biopsies were processed as previously described [9]. In brief, 90nm sections were mounted on 200 mesh copper grids before double staining with uranyl acetate and lead citrate. Grids were then examined using a transmission electron microscope (Philips CM10, Eindhoven, The Netherlands). Micrographs were recorded on Kodak 4489 electron microscopy film (KODAK, NJ, USA) and printed for review and reporting.

#### Confocal Laser Scanning Microscopy

Microscopic examination was performed on biopsy specimens using CLSM imaging with a Nikon A1Si confocal microscope using 40x and 60x Plan Apo objectives.

### Evaluation of middle ear mucosal specimens with pathogen-specific probes

**16S rRNA FISH - **Biopsies were incubated for three min at room temperature in 50%, 80% and 100% ethanol, respectively. Each biopsy was incubated at 37°C in hybridisation chambers with 50 μl of 10 mg/ml of lysozyme in 0.1-M Tris and 0.05-M Na_2 _EDTA and washed with sterile PBS.

Bacterial presence, biofilm structure and multi-species interactions were visualised using FISH and CLSM. Hybridisation probes were optimised for *S. pneumoniae*, nontypeable *Haemophilus influenzae*, *Moraxella catarrhalis *and *Staphylococcus aureus *as well as a universal eubacterial (EUB338) probe. Middle ear mucosal biopsies from all 18 children were examined for evidence of biofilm or intracellular infection using CLSM following FISH and generic nucleic acid labelling with Hoechst 33342. As mucosal biopsy size was a limiting factor, with samples being on average < 1 mm^3^, only a single set of two bacterial species specific FISH probes could be performed on each specimen. In addition the universal EUB338 probe was used on all specimens for the specific detection of bacteria.

FISH was conducted as previously described by Hall-Stoodley *et al *[8] using 16S rRNA probes labelled with AlexaFluor 488, 546 or 633 dyes (Invitrogen Technologies) and incubated overnight in 1.2 ug/ml Hoechst 33342 (Invitrogen Technologies) to stain nuclei. Biopsies were mounted in low fade mounting media and imaged using four colour CLSM. Probes were selected for hybridisations based on bacterial culture and PCR results from MEE. If MEE was not present or if the sample was culture and/or PCR negative, probes for the most prevalent organisms were used (*S. pneumoniae*, *M. catarrhalis *or *H. influenzae*). The following probes were used: EUB338 for the domain bacteria (with a 5'-3' sequence, GCT GCC TCC CGT AGG AGT) [23], *S. pneumoniae *probe (GTG ATG CAA GTG CAC CTT) [24], nontypeable *H. influenzae *(CCG CAC TTT CAT CTT CCG) [25], *S. aureus *(GAA GCA AGC TTC TCG TCC G) [24] and *M. catarrhalis *(CCG CCA CUA AGU AUC AGA) [8]. Specificity of 16S rRNA probes was extensively tested and validated using ATCC and clinical isolates (data not shown).

### Image analysis and interpretation

As there is little data on characterisation and definitions of biofilms in middle ears of children, definitions by Hall-Stoodley et al [8] and from the chinchilla model of biofilm [5] were used as guides to assess and characterise bacterial presence in these specimens. Samples were scored as being positive for biofilm presence when characteristic biofilm morphology was apparent and included presence of aggregated bacteria (often in the form of microcolonies), usually adherent to a surface and surrounded by extracellular matrix [5,8]. When images were suggestive of biofilm these were further evaluated using high resolution electronic zoom with samples being recorded as positive when results demonstrated bacterial morphology, using size (approximately 0.5-2 μm) and shape (either cocci or cocco-bacilli), biofilm structure and fluorescence with appropriate signal. No attempt was made to quantify biofilm formation. Bacteria were marked as being intracellular when there was close association of bacteria (as demonstrated by size, morphology and appropriate fluorescent signal) with intact host cell nuclei, as described by Zautner *et al*, [26]. When intracellular location was suggested, images were assessed by examining individual focal planes to ensure true association of bacteria with host nuclei.

## Results

### Patient characteristics

General patient characteristics are described in Table 1 and 2. Twenty middle ear biopsies were obtained from 20 children undergoing ventilation tube insertion. Of these, 6 (30%) had a diagnosis of COME only, 6 (30%) had a diagnosis of rAOM only, and 8 (40%) had both diagnoses. Eleven (55%) were girls and the median age was 4.3 yr (range, 1.2-9.9). Control biopsies were obtained from a 1.6 year old female undergoing cochlear implantation due to sensorineural hearing loss. All children in this group identified as Caucasian. Six of the 20 children with OM had been previously vaccinated with the 7-valent pneumococcal conjugate vaccine, as had the child undergoing cochlear implant surgery. The pneumococcal vaccination status for two children (No's 2 and 19) were unknown. Two children were receiving cephalosporin antibiotics at the time of surgery (Table 2) and one other child (Child 7) had received amoxycillin in the month prior to surgery.

**Table 1:** Gender, age, diagnosis and microscopic evidence of biofilm and intracellular bacteria in middle ear mucosal biopsies using Transmission Electron Microscopy.

**Child No**.	Sex (Age, yr)	Diagnosis	Biofilm	Intracellular bacteria
1	F (1.2)	RAOM and OME	-	-

2	M (2.1)	RAOM and OME	-	+

3	M (5.2)	RAOM	-	+

**Table 2:** Gender, age, diagnosis, species presence and microscopic evidence of biofilm and intracellular bacteria using Fluorescence *In Situ *hybridisation (FISH) in middle ear mucosal biopsies and effusions assayed using culture, PCR and FISH methods for pathogen detection.

					PCR		FISH						
									
**Child No**.	Sex (Age, yr)	Ear	Diagnosis	Current Abx	Culture	Spn	EUB	Spn	Hi	Mcat	Sau	Biofilm	Intracellular bacteria
**Children with recurrent acute otitis media**													

4	F (1.4)	R	RAOM	N	-	-	+	na	+	+	na	+	+

5	M (1.8)	L	RAOM	N	na	na	+	-	+	na	na	-	+

6	F (3.2)	L	RAOM	N	na	na	+	na	+	-	na	+	+

7	M (6.1)	L	RAOM	N	+ mixed	+	+	+	na	-	na	-	+

8	F (9.3)	L	RAOM, Cholesteatoma	N	na	na	+	-	na	+	na	-	-

**Children with recurrent acute otitis media and otitis media with effusion**													

9	M (1.4)	R	RAOM, OME	Y	-	-	+	na	-	+	na	+	+

10	M (1.8)	L	RAOM, OME	N	+ Spn, Mcat	+	+	+	na	+	na	+	+

11	F (1.8)	L	RAOM, OME	Y	-	+	+	-	+	na	na	-	+

12	F (3.5)	L	RAOM, OME	N	-	-	-	na	-	-	na	-	-

13	M (7.5)	L	RAOM, OME	N	-	-	+	na	+	+	na	+	+

14	F (8.9)	L	RAOM, OME	N	na	na	+	+	-	na	na	+	+

**Children with otitis media with effusion**													

15	M (1.5)	L	OME	N	na	na	+	-	-	na	na	+	-

16	F (5.4)	L	OME	N	+ CNS	-	+	na	-	+	na	+	+

17	F (6.0)	L	OME	N	+ Sau	-	+	na	na	-	+	+	+

18	F (6.3)	R	OME	N	na	na	-	-	na	-	na	-	-

19	F (8.0)	R	OME	N	na	na	+	-	-	na	na	+	-

20	M (9.9)	R	OME	N	na	na	+	+	na	+	na	+	+

**Control cochlear implant**													

21	F (1.6)	L	Sensorineural hearing loss	N	na	na	-	-	-	na	na	-	-

Positive from number tested (not including healthy control)				2/17	4/9 (44%)	3/9 (33%)	15/17 (88%)	4/10 (40%)	5/11 (45%)	7/12 (58%)	1/1	11/17 (64%)	12/17 (71%)

Biopsies are from children undergoing ventilation tube insertion for COME and recurrent AOM as well as a negative control from a child with no history of middle ear disease undergoing cochlear implantation surgery.

Abbreviations: EUB - eubacterial probe, Spn - *S. pneumoniae *(organism and probe), Hi - *H. influenzae *(organism and probe), Mcat - *M. catarrhalis *(organism and probe), Sau - *S. aureus *(organism and probe), CNS - Coagulase negative staphylococci (organism). na - Not available.

### Microbiological findings in MEE

No MEEs were collected from those children for whom biopsies were examined using TEM (Table 1).

MEEs were collected from 9 of the 20 ears from which biopsies were taken. MEE was not present in all ears since several children were recruited between AOM or OME episodes. They were assayed via culture for respiratory bacterial pathogens and by PCR for the Ply gene for the presence of *S. pneumoniae *(Table 2). Of the 9 MEE cultured, one was positive for *S. pneumoniae *and *M. catarrhalis*, one was positive for *S. aureus*, one for Coagulase negative staphylococci and one was positive for unspecified mixed bacterial flora (not containing any middle ear pathogens). The Ply PCR was positive in three samples. Of these 3 Ply positive samples one was culture positive for *S. pneumoniae*, one was culture negative for any bacteria and the third sample was culture positive for mixed bacterial flora not including any of the middle ear pathogens. All remaining samples were Ply PCR negative.

### Evidence of biofilms and intracellular infection

**Figure 1:**
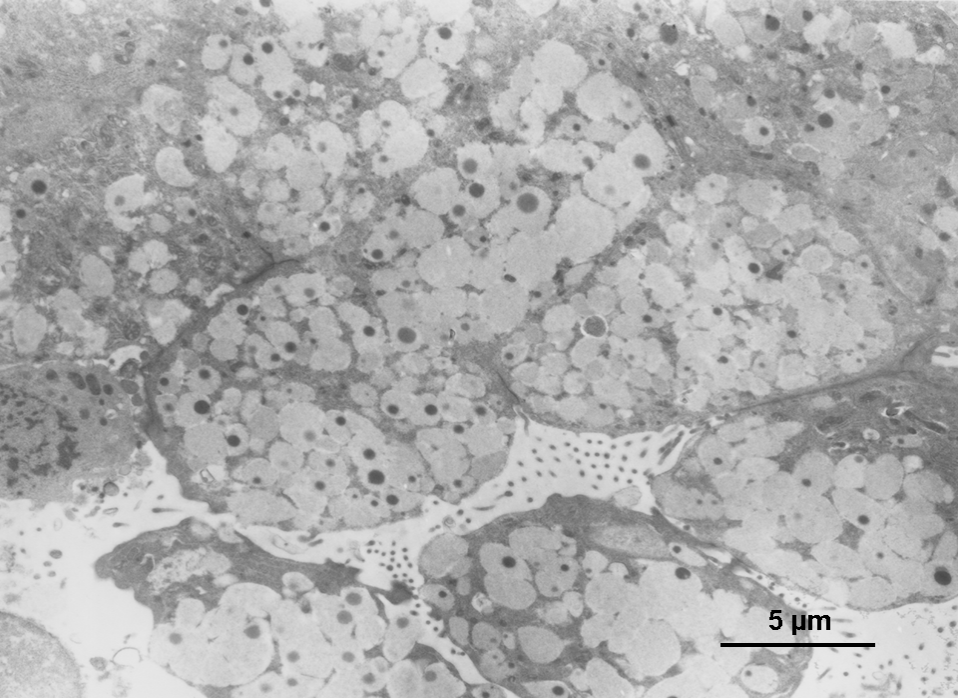
**Representative TEM of the middle ear mucosa from a child with a history of COME and recurrent AOM**. This biopsy was from a 25 month old male, MEE was not collected. Image shows mucus-secreting epithelial cells (12-15 μm) overwhelmed with coccal bacteria in mucus containing vacuoles.

Two of the 3 samples examined using TEM were seen to have intracellular bacteria in mucus containing vacuoles within the epithelial cells (Table 1 Figure 1). No biofilm was observed using TEM.

**Figure 2:**
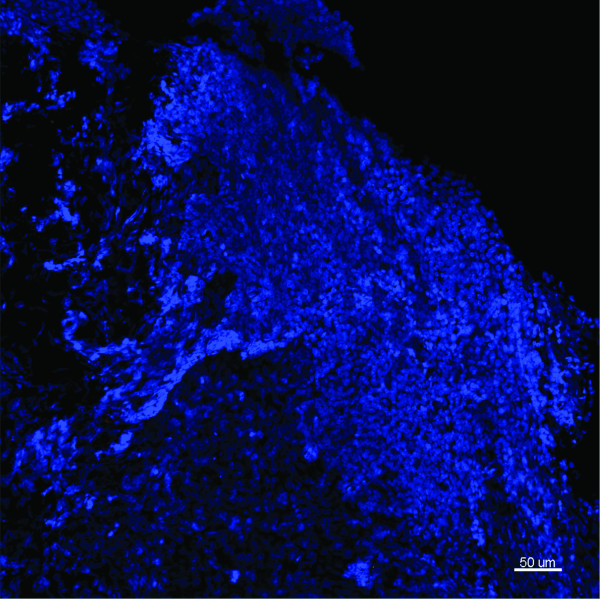
**Representative image from the middle ear mucosal biopsy of a child having a cochlear implant who has no history of middle ear disease**. Child 21. FISH -EUB338 (Yellow), *S. pneumoniae *(green), *H. influenzae *(pink) and Hoechst 33342 (nuclei stain - blue). This maximum intensity projection (Z = 20.5 μm) demonstrates the normal mucosal tissue with no evidence of bacteria. Scale bar = 50 μm.

**Figure 3:**
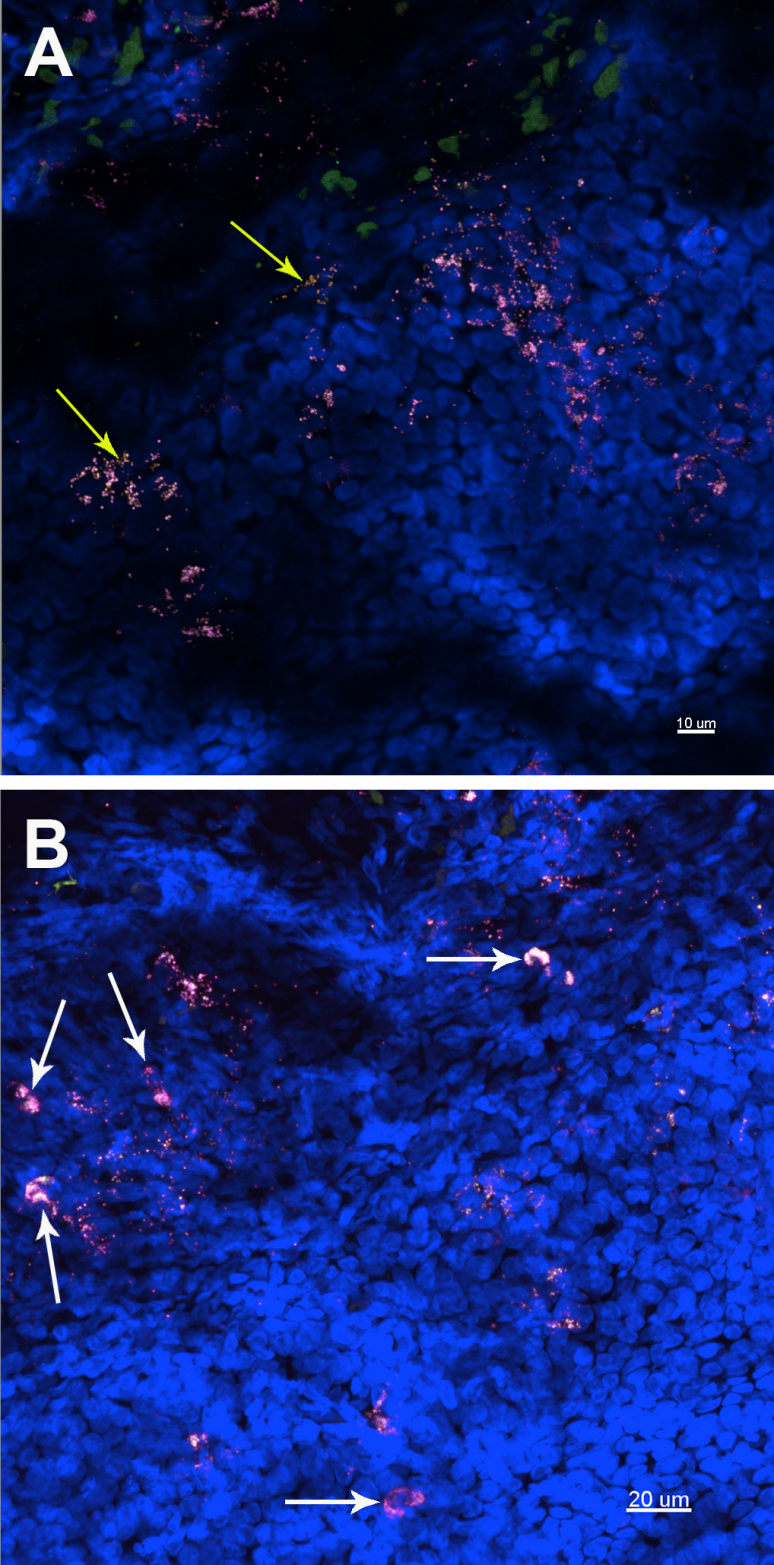
**Representative images of a mucosal biopsy from a child suffering with rAOM**. Middle ear fluid culture and Ply PCR negative, showing biofilm and intracellular infection. Child 4. FISH: EUB338 (yellow), *M. catarrhalis *(green), *H. influenzae *(pink) and Hoechst 33342 (nuclei stain - Blue). A) *H. influenzae *is the predominant pathogen, with other non-identified bacteria also evident (yellow arrows). Maximum projection image (Z = 29 μm), scale bar = 10 μm. B) Intracellular *H. influenzae *are apparent throughout the tissue as nuclei associated bacterial clusters (white arrows), as well as in biofilm. Maximum projection image (Z = 40 μm), scale bar = 20 μm.

**Figure 4:**
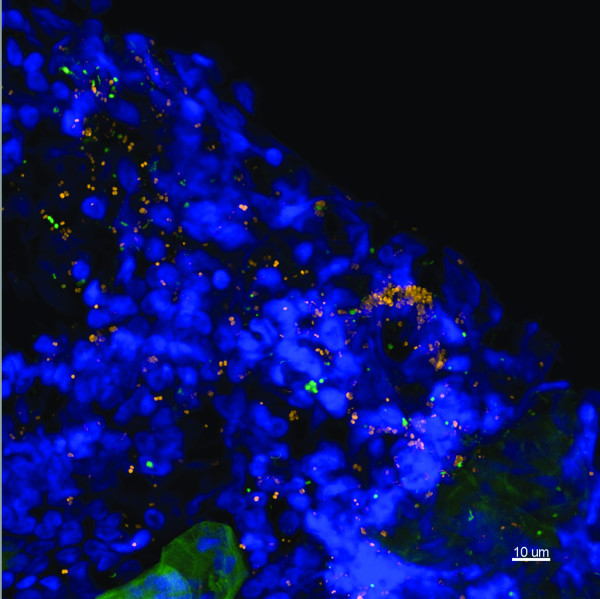
**Representative image of a mucosal biopsy from a child suffering from rAOM and COME**. MEE was not present. Child 14. FISH probes included EUB338 (yellow), *S. pneumoniae *(green), negative for *H. influenzae *(pink), Hoechst 33342 (nuclei stain - blue). This is a maximum intensity projection (Z = 39 μm) showing multispecies biofilm covering the mucosa. The biofilm is seen to consist of *S. pneumoniae *and other unidentified bacteria. These bacteria are also interspersed within the tissue. Scale bar = 10 μm.

**Figure 5:**
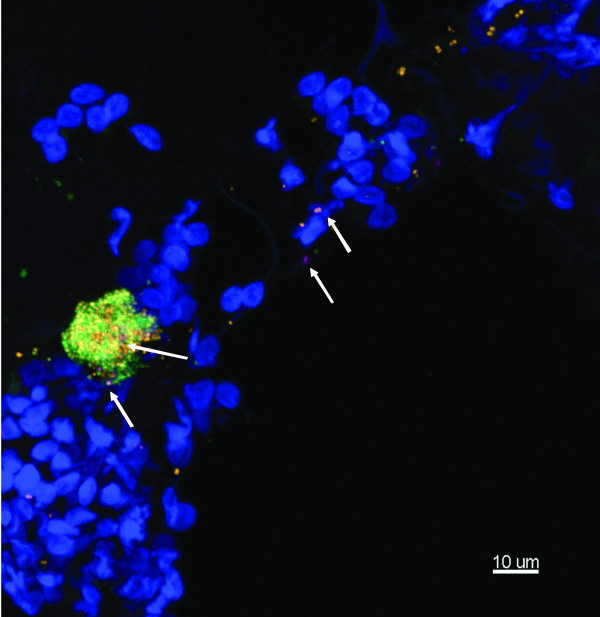
**Representative image of a mucosal biopsy from a child with a history of COME**. No middle ear fluid was taken. Child 20. FISH - EUB338 (Yellow), *M. catarrhalis *(green), *S. pneumoniae *(pink) and Hoechst 33342 (nuclei stain - blue). Maximum intensity projection (Z = 19.5 μm) showing microcolony formation, mainly *M. catarrhalis *and *S. pneumoniae *(arrows) scattered throughout the mucosa. Scale bar = 10 μm.

Subsequently, 17 biopsy samples were examined using FISH and CLSM. Depending on biopsy size and the presence of bacteria, between 2 and 9 stacks of images were taken and examined for each specimen, representative maximum projections were presented. Universal bacterial EUB338 hybridisation was demonstrated in 15 of the 17 specimens (88%) (Table 2). Using 3 different probes for *H. influenzae, S. pneumoniae *and eubacterial 16S rRNA genes, no bacteria were observed in two biopsies taken from the same ear of a control cochlear implant case (Figure 2). Overall 11 (65%) of the 17 middle ear mucosal biopsies were positive for bacterial biofilm based on morphological and fluorescence criteria. Of these 11, 9 were also positive for intracellular bacteria demonstrating discrete aggregates of bacteria closely associated with host nuclei. Twelve (71%) of the 17 samples were positive for intracellular bacteria. Two samples showed scant bacteria which were scattered throughout the biopsies, without characteristics of biofilm or intracellular infection. Similar rates of bacterial biofilm and intracellular bacteria were seen in children with rAOM (4 out of 5 samples positive), COME (5 out of 6 samples positive) or both diagnoses (5 out of 6 samples positive; Table 2). Biofilm morphologies varied, ranging from microcolonies to large bacterial clusters, however no differences were observed between diagnostic groups (Figures 3, 4 and 5). Intracellular bacteria were frequently present throughout the positive specimens.

When MEE was present and culture was positive, bacteria were identified by CLSM and FISH in all cases. For the MEE sample culture positive for *M. catarrhalis *and *S. pneumoniae *and PCR positive for *S. pneumoniae*, FISH was also positive for these bacteria. The MEE that was culture positive for *S. aureus *had a corresponding middle ear mucosal biopsy which was positive for *S. aureus *by FISH. This sample was negative for the additional *M. catarrhalis *probe but positive for other unidentified bacteria using the eubacterial probe.

Five of 11 biopsies (45%) assessed for *H. influenzae *by FISH were positive, including 3 of 3 rAOM and 2 of 5 rAOM/COME specimens (Table 2). None of the 3 COME only specimens tested were positive for *H. influenzae *by FISH. FISH identified *S. pneumoniae *in 4 of 10 biopsies (40%), including 1 of 3 rAOM, 2 of 3 rAOM/COME specimens and 1 of 4 children with COME only. *M. catarrhalis *was examined in 12 mucosal biopsies and of these 7 (58%) were positive. Two of 4 biopsies positive for *M*. *catarrhalis *were from children with rAOM only and 3 of 4 positive specimens were from children with both rAOM and COME.

In 4 of the 5 *H. influenzae *positive samples, this pathogen was observed intracellularly only. In one of these 5 samples *H. influenzae *was found in biofilm and intracellularly concurrently (Figure 3). *S. pneumoniae *and *M. catarrhalis *were shown to reside both intracellularly and in biofilm structures.

## Discussion

Initial examination of middle ear mucosal biopsy samples using TEM indicated the presence of intracellular bacteria in mucus containing vacuoles, based on morphology and electron density. This mucus may be exocytosed onto the mucosal surface, contributing to the persistence of effusion as we have previously suggested in biopsies from children with COME [9]. Using TEM biofilm was not detectable in samples from children with rAOM, similarly to what was observed in children with COME [9] and, bacterial species were not able to be determined using this technique. It is apparent that while TEM may be a suitable technique to demonstrate intracellular bacteria within mucosal biopsies, it is less useful in examining biofilm presence or in determining bacterial species within a specimen. We therefore used FISH and CLSM. CLSM allowed us to examine hydrated specimens and the use of FISH allowed determination of bacterial structures and the species within these.

Using FISH and CLSM we have demonstrated that multiple bacterial species including pathogens associated with acute OM, are present intracellularly and/or in biofilm in the mucosa of 82% of children undergoing ventilation tube insertion for COME and rAOM. Separately, 64% of biopsies were positive for biofilm, while 71% were positive for intracellular bacteria. No difference was observed between children with either rAOM or COME, with biofilm and intracellular bacteria being demonstrated at similar rates in both. These findings are similar to those described by Hall-Stoodley *et al*, the only study using FISH to evaluate biopsies from a comparable cohort, showing bacterial clusters containing otopathogens, on the middle ear mucosa of children with both rAOM and COME [8]. Importantly, at least one of the known OM pathogens was demonstrated in 12 of the 14 samples positive for biofilm or intracellular infection, and no bacteria were seen on the healthy control middle ear mucosa. These findings support the hypothesis that the bacterial OM pathogens are present either intracellularly or in bacterial biofilms or both. The presence of otopathogens may play an important role in OM pathogenesis and could be a source of chronic inflammation and/or recurrent infection.

*H. influenzae *was found in 45% of biopsies, similar to levels detected in the MEEs of Australian children undergoing surgery for rAOM (47%) by PCR [27]. However, rates are lower than those detected by Hall-Stoodley et al who detected 70% of MEEs were positive for *H. influenzae *by PCR and 7 of 7 biopsies positive using FISH. This may reflect population differences or may reflect the differences in methodology. As the microbiology of our samples were unknown we chose to follow guidelines outlined by Hugenholtz *et al *for fixing specimens containing both Gram-positive and Gram-negative organisms [28]. All samples were fixed in paraformaldehyde. Furthermore we also pre-treated all biopsies with lysozyme which is recommended to permeabilise Gram-positive organisms. While this may affect the Gram-negative organisms and may be responsible for the differences observed between our study and that of Hall-Stoodley *et al *[8], during assay optimisation this was demonstrated to have no affect on hybridisation rates or on the bacterial morphology. We therefore believe this to be a true representation of *H. influenzae *presence in these biopsies.

Multiple bacterial species were present in all specimens suggesting that OM is a polymicrobial infection. For example, *S. pneumoniae *and *M. catarrhalis *were found in the same specimen in two of five cases (40%). *M. catarrhalis *and *H. influenzae *were found together in two out of six specimens (33%) tested. This may be important clinically as evidence in animal models suggests that in a polymicrobial biofilm bacterial species are able to confer protection from host defences and antimicrobials to each other and thus more resistant that single-specie biofilms [29]. Furthermore, in the nasopharynx of mouse OM models, bacterial composition and viral infection are shown to have significant effects on OM incidence and severity [30]. Although *S. pneumoniae *and *H. influenzae *have been described to reside together in the middle ear of children with OM [8,31], interestingly these pathogens were never observed together in the same specimen (0 of 5 specimens) in our study. While the number of samples tested for these species was small, our findings may reflect the competitive interactions observed between *S. pneumoniae *and *H. influenzae *[32-34]. In a significant proportion of biopsy samples, multiple unidentified bacterial species were observed. These bacteria were often not cultured even when shown to be present using FISH or PCR. Other fastidious organisms such as *Alloiococcus otitidis *may also play a role in this disease as they have been found using PCR and culture in children with both OME and rAOM [35,36].

Using TEM it is clear that intracellular bacteria were present within the epithelial cells of biopsies assessed in children with rAOM, similar to those seen in the middle ear mucosa of children with COME [9]. Biopsy samples using FISH were assessed as having intracellular bacteria when there was close association of bacteria with intact host cell nuclei, as described [26]. These results were consistent with TEM findings. In animal OM models, biofilm is often shown to be patchy [5] and may be missed during sampling or disruption of these structures during processing [8,37]. This differs for intracellular bacteria which are more likely to be embedded within the biopsy and thus less likely to be dislodged or affected by processing techniques. We believe it is for these reasons we observed intracellular bacteria more often than bacterial biofilms and that they may represent continuums of the same disease process. In each of the cells, intracellular bacteria belonged to the same species. This may reflect the formation of intracellular "biofilm pods" similar to those formed within bladder epithelial cells by uropathogenic *Escherichia coli *[38-40]. These are important as in these intracellular compartments bacteria are able to proliferate and persist, subverting host immune responses and increasing resistance to antimicrobial treatments [10,12,41].

At the time of this study, PCRs for pathogens other than *S. pneumoniae *were not available in our laboratory. It has also since become apparent that not all strains of *S. pneumoniae *contain a functional Ply gene and as such these bacteria may not always be detected when using this PCR. Therefore the selections of probes to use on the biopsy specimens based on MEE PCR results were not optimal and represent limitations of this study. By using the eubacterial probe and combinations of probes for the most common otopathogens, we could successfully determine bacterial presence in either biofilm formation or intracellularly. While only one healthy mucosal specimen was available, on thorough examination no bacteria could be identified; this is in accordance with another study [8] which demonstrated that no bacteria were evident in healthy middle ear mucosal samples. Little is known regarding the progress of OME and rAOM. It is difficult to determine whether biofilm and intracellular infection are a reflection of the residual stage on the way to clearance of the infection or whether they represent the main mechanisms of persistence within the host. Whether the otopathogenic bacteria within these intracellular compartments demonstrate biofilm phenotypes is currently unknown and requires further investigation. Given the severity and chronic nature of infection in many of these children, it is clear however that these infections do not resolve readily on their own and that biofilm and intracellular sequestration represent ways in which bacteria can resist both host defences and current antimicrobial treatments.

Otopathogenic bacteria are known to form biofilms *in vitro *and in animal models [5], are able to invade and survive within cells *in vitro *[10-15] and in adenoidal cells [16,17]. While biofilm and intracellular infection with pathogenic species are known to play roles in the chronicity of many diseases [42-45] very few studies exist demonstrating the presence of biofilm and/or intracellular infection in the middle ear mucosa of children with chronic or recurrent OM. A more recent study examining MEE from children with COME detected no biofilm [20]. These differences may reflect the use of MEE smears rather than biopsies.

To our knowledge this is the first study that has concurrently identified both bacterial biofilm and intracellular bacterial sequestration of known otopathogens in the middle ear mucosa of children with COME and rAOM. Successful identification of otopathogenic species in biofilms or intracellularly may encourage the development of treatments that target both bacterial biofilm and intracellular infection, since both might contribute to persistence and recurrence of infection. Current treatment regimens with β-lactam antibiotics (including amoxycillin) which do not target intracellularly sequestered pathogens may be less effective than macrolide or fluoroquinolone class antibiotics which have been shown to penetrate and concentrate intracellularly depending on antibiotic resistance patterns. Studies examining the effectiveness of combination antibiotic treatment for rAOM and COME are urgently required to try and improve the outcome of non surgical interventions. Bacteria within biofilms and located within cells may also be less susceptible to killing by naturally acquired or vaccine-induced antibody which contributes to the recurrent nature of this disease. Investigational treatments specifically for biofilm diseases and those targeting intracellular bacteria may play an important role in OM management in the future.

## Conclusion

CLSM is a more robust imaging method to determine biofilm presence in middle ear biopsies when compared to TEM. Using FISH and CLSM, bacterial biofilm and/or intracellular infection with known otopathogens have been demonstrated on the middle ear mucosa of children with COME and rAOM. While the role of biofilm and intracellular infection in both of these disease presentations remains to be determined, these infection patterns may help explain the ineffectiveness of current treatments in preventing recurrent episodes. While larger studies on which quantitative analyses could be performed would be useful in the future this highlights the need for alternative strategies to target both biofilm and intracellular infection in the middle ear mucosa. Future studies looking at the mechanisms of bacterial persistence in high risk groups such as Indigenous Australians would be important in understanding these disease processes and to design more effective treatment and prevention.

## Competing interests

R.B. Thornton has received travel funding from GlaxoSmithKline.

S.P Wiertsema has received Institutional funding from GSK for investigator-led epidemiological studies in otitis media and has received travel support from GSK

H.L. Coates and S. Vijayasekaran consult for GlaxoSmithKline Biologicals.

P.C. Richmond has received Institutional funding from GSK for investigator-led epidemiological studies in otitis media and has received travel support from GSK, Wyeth and other vaccine companies to present scientific data and chair workshops. He has no shares, paid employment, or consultancies with any pharmaceutical companies.

Other authors have no competing interests to declare.

## Authors' contributions

RT was involved in original study design, participant recruitment, sample processing, designed and conducted the fluorescent imaging experiments, and drafted the manuscript. PF, conducted the electron microscopy. PCR co-ordinated original study design and experimental design. SW, JL, HC & SV were involved with recruitment and sample collection. AK managed bacterial culture and PCR assays. PJR co-ordinated experimental design. All authors have revised and approved the final manuscript.

## Pre-publication history

The pre-publication history for this paper can be accessed here:

http://www.biomedcentral.com/1471-2431/11/94/prepub
